# Synergistic application of cardiac sympathetic decentralization and comprehensive psychiatric treatment in the management of anxiety and electrical storm

**DOI:** 10.3389/fnint.2013.00098

**Published:** 2014-01-02

**Authors:** Sahib S. Khalsa, Leila Shahabi, Olujimi A. Ajijola, Alexander Bystritsky, Bruce D. Naliboff, Kalyanam Shivkumar

**Affiliations:** ^1^Department of Psychiatry, Semel Institute for Neuroscience and Human Behavior, David Geffen School of Medicine, University of CaliforniaLos Angeles, CA, USA; ^2^Department of Medicine, UCLA Cardiac Arrhythmia Center, David Geffen School of Medicine, University of California Los AngelesLos Angeles, CA, USA; ^3^Department of Medicine, Oppenheimer Family Center for Neurobiology of Stress, David Geffen School of Medicine, University of California Los AngelesLos Angeles, CA, USA

**Keywords:** autonomic nervous system, stellate ganglion, anxiety disorders, implantable cardioverter defibrillator, electrical storm, interoceptive awareness, PTSD, cognitive behavioral therapy

## Abstract

We report here, for the first time, two cases demonstrating a synergistic application of bilateral cardiac sympathetic decentralization and multimodal psychiatric treatment for the assessment and management of anxiety following recurrent Implantable Cardioverter Defibrillator (ICD) shocks. In a first case the combination of bilateral cardiac sympathetic decentralization (BCSD), cognitive behavioral psychotherapy and anxiolytic medication was sufficient to attenuate the patient's symptoms and maladaptive behaviors, with a maintained benefit at 1 year. Among the more prominent subjective changes in the post-lesion state we observed a decrease in aversive interoceptive sensations, particularly of the heartbeat. The patient continued to experience cognitive threat appraisals on a frequent basis, although these were no longer incapacitating. In a second case, we report the effect of BCSD on autonomic tone and subjective state. In the post-lesion state we observed attenuated sympathetic responses to the valsalva maneuver, isometric handgrip and mental arithmetic stressor, including decreased systolic and diastolic blood pressure and, decreased skin conductance. Collectively, these preliminary findings suggest that an integrative, multidisciplinary approach to treating anxiety disorders in the setting of ventricular arrhythmias and recurrent ICD shocks can result in sustained improvements in physical, psychological, and functional status. These findings raise the possibility of a potential role for the stellate ganglion in the modulation of emotional experience and afferent transmission of interoceptive information to the central nervous system.

## Background

Implanted Cardioverter Defibrillator (ICD) placement generally occurs when a patient with certain types of cardiovascular disease, including cardiac arrhythmia, structural heart disease, heart failure or history of Sudden Cardiac Arrest (SCA), is judged to be at increased risk of fatal arrhythmias without intervention (Epstein et al., 2008; Tracy et al., 2012). Although this intervention has a well demonstrated survival benefit (Moss et al., 1996; Maron et al., 2000; Corrado et al., 2003), it is associated with a host of psychological sequelae in pediatric and adult populations that are often incompletely addressed at the time of implantation (Sears et al., 1999, 2009, 2011; Sears and Conti, 2002; Hazelton et al., 2009; Dunbar et al., 2012). The most significant among these appears to be the occurrence of repeated and unpredictable defibrillator shocks, whether appropriate (i.e., discharge in the presence of a detected arrhythmia; occurring in 17–64% of patients) or inappropriate (i.e., discharge without presence of detected arrhythmia; occurring in 10–24% of patients) (Sears and Conti, 2003; Germano et al., 2006).

ICD discharge, whether appropriate or inappropriate, poses a particularly challenging psychological event that can condition patients to become fearful in their search for an explanation. Prominent features include the maladaptive development of hypervigilance toward interoceptive cues, fear and avoidance of interoceptive sensations, avoidance of physical and mental situations that might trigger interoceptive sensations, and attachment to objects or people perceived to assure safety (i.e., safety seeking). This cycle of events, which we have described previously (Bystritsky et al., 2013), drives psychiatric comorbidity and contributes to disorders such as Panic Disorder, Post-Traumatic Stress Disorder, and Social Anxiety disorder, sometimes all within the same individual. Ultimately, between 11 and 38% of these patients are estimated to develop anxiety disorders and between 11 and 46% to develop major depression (Magyar-Russell et al., 2011; Dunbar et al., 2012).

Although anxiety related to ICD implantation and discharging may be mild or transient, for some patients it can lead to long lasting debilitating behavioral changes. For example, one cardiomyopathy patient began spending all of his time in his local hospital after receiving recurrent ICD shocks, under the rationale that this was the safest place to be to avoid further harm. He remained in these areas of the hospital for several months and experienced significant fear upon leaving. Due to continued worry about being shocked, the patient even obtained a stethoscope in order to better listen to his heartbeat. When he noticed his heart racing he would auscultate it with the stethoscope, claiming he could distinguish between normal tachycardia and tachycardia associated with his arrhythmia, and predict the onset of a shock approximately 3 seconds later. Another patient refused to shower alone for 3 months, out of fear that he would be shocked by his ICD, become incapacitated, and no one would find him. This same patient remained within a three mile radius of his home for 1 year, because it was located near a hospital, for fear that he would be shocked and would not be able to be evaluated and resuscitated in time.

These patient experiences and the overall psychological burden of ICDs suggest that a comprehensive assessment and treatment plan involving medical and psychiatric intervention is warranted, particularly for the most severely affected individuals. We report here, for the first time, the results of a synergistic application of bilateral cardiac sympathetic decentralization (BCSD) and multidimensional psychiatric treatment resulting in successful management of anxiety following ICD multiple shock storm. We present a detailed description of the physical and psychological responses to BCSD with up to 1 year of follow up, in two patients. We examined the effect of BCSD on autonomic tone in both patients, using specific tasks shown to directly activate the medial visceromotor network, including the anterior cingulate cortex (ACC) and medial prefrontal cortex (King et al., 1999; Critchley et al., 2000; Wong et al., 2007; Gianaros et al., 2012; Shoemaker et al., 2012). However, as we were unable to obtain pre-lesion data from one patient (Case 1), we report only the effects of such testing before and after BCSD in the other patient (Case 2).

To measure shock related cognitive threat appraisals we adapted a scale from the pain literature, the Pain Catastrophization Scale (Sullivan et al., 1995). This scale was designed to measure several cognitive distortions associated with chronic pain including magnification, rumination, and helplessness. We adapted this scale as we believe these phenomena are commonly present during the experience of chronic anxiety related to painful and distressing ICD shocks, and have renamed it the Shock Catastrophization Scale (SCS) (see **Appendix** for full scale details including reliability analysis).

## Case 1

### History of present illness

The patient was a 38 year-old healthy male without a history of cardiovascular or psychiatric disease who experienced a sudden collapse while bicycling to work. On primary assessment by paramedics he was found to be pulseless. Cardiopulmonary resuscitation was initiated. He was electrically cardioverted, resuscitated, and rushed to the nearest hospital. To preserve cerebral function, the patient was placed in an induced hypothermic and comatose state. During his recovery he experienced an episode of ICU delirium with agitation resulting in administration of sedative medication. After recovering from this delirium, he reported discrete retrograde memory loss from 1 day prior to the event through the first week after his SCA. He underwent ICD implantation (dual lead, atrial and ventricular, Medtronic Inc. St Paul, MN) at 2 weeks for secondary prevention of sudden death, and was discharged from the hospital shortly afterwards. He returned to work 3 weeks after the event. Immediately after ICD implantation the patient described being very fearful of being alone in the house with his young children. In response, he and his wife developed procedures to ensure that their children knew how to call emergency medical services if he were to become unresponsive or be shocked.

The patient experienced his first ICD shock 11 months later, while riding the exercise bike at a local gym. He received a total of 6 shocks over approximately 2–3 min. After realizing he was being shocked, he did not recall experiencing anxiety. Rather, he described responding calmly and without terror, in a way that did not distract or alert others to his experience (i.e., no social disruption). In the patient's words: *“they had told me it would feel like getting kicked in the chest by a horse. It didn't really feel like that. It was just a very loud, like a ‘thunk!’ sound. I maintained consciousness. At first I wasn't sure what it was. By the time the second one came, I realized this must be the defibrillator going off. And I was conscious the whole time, and walking. I don't know what the interval was, probably I guess 15–20 seconds in between [each shock].”* Patient's wife: *“you weren't very fazed by it though, and nobody else knew what was happening.”* Patient: *“Right. And that goes to some of my anxiety, and why some of the subsequent shockings impacted me more than that one. This one I was able to keep private. Nobody knew what was happening. I knew I got shocked. I went home, called my doctor. It wasn't a very big public scene.”*

The patient experienced his second episode of repeated ICD shocks while at the gym, after 15 min of jogging on the treadmill. He received a total of 7 shocks approximately 20 s apart. He again denied experiencing any social disruption, but this time felt terror over the lack of control over his symptoms. He also described experiencing physical pain with the shock. In the patient's words: *“It's not so much the pain of the shock as much as, is there another one coming? Is this the last one, or am I bracing for another one? I'm trying to calm down, to get my heart rate down, but it's almost impossible because I'm under this utter terror that another one is coming, and another one, and is it ever going to stop?”*

During this second set of shocks, despite the reported fear and aversive anticipation of subsequent shock, he denied experiencing thoughts of death. When asked how he considered the meaning of the shocks, he stated *“I'm not afraid I'm going to die, I don't feel like this event is going to kill me. At that time I'm more concerned with ‘will it stop? How can I make it stop, and who is going to help me make it stop?’ I feel somewhat powerless in this whole sequence of being subjected to these shocks, with really no ability to make it stop at some point. That's what impacts me most when it's happening.”* He further noted: *“When I got my defibrillator originally, I remember this distinctly, my doctor told me ‘You’re gonna die some day. But it's not going to be from this. You've got the defibrillator now, you're not going to die from this.' And I certainly took some comfort from that.”*

After uploading data from the ICD to his cardiologist, which demonstrated a ventricular tachycardia progressing to ventricular fibrillation, he was referred for further treatment via cardiac catheter ablation procedure to target a focus of premature ventricular contractions (PVCs) that had been previously identified. He also underwent an atrial lead revision and generator change during a separate procedure.

After the second set of shocks, the patient became preoccupied with the potential impact the shocks might have on his social milieu, a fear that *“I'm gonna get shocked in front of other people and they will think I'm defective.”* He developed several avoidance behaviors. He stopped exercising or engaging in all activities that could potentially increase his heart rate (including sex), under the belief that this would cause him to experience another shock. Hypervigilance emerged: he began scanning his body for signals that could predict a shock, such as feeling a fast heartbeat, or a *“not good”* feeling. He began to seek further reassurance that he was unlikely to receive another shock, by frequently contacting his cardiologist and frequently monitoring his ICD's electronic output. During each of these visits, no abnormalities were found.

Two and a half years after receiving his first shock, the patient reported being shocked *“in a different way.”* While out of town and walking with some co-workers, he received a shock and fell to the ground, grunting and groaning. There was no loss of consciousness. He continued to receive a total of 8 shocks in a row. Paramedics arrived at the scene and administered anxiolytic benzodiazepines, which the patient believes aborted the shocks. He was hospitalized for 4 days. Interrogation of his ICD revealed he had received appropriate ICD discharges in response to the spontaneous development of ventricular tachycardia. There were no further abnormal findings on electrophysiological exam, and he was discharged without application of any specific procedures or medications. This experience affected the patient differently, because *“I could no longer hide it. There's shame involved, [that] there's something wrong with me.”* The lack of specific treatments rendered further anxiety. Riding home alone on the airplane he was extremely anxious about being shocked again, without access to emergency medical services. He experienced his first panic attack in midair, with prominent dyspnea, palpitations, dizziness, and fear of losing control over his mind and body. He also worried that being shocked in front of other people would cause them to think he was defective. After arriving home safely, he began to experienced recurrent panic attacks, approximately 10–15 times per month. These were often provoked when in open or crowded spaces, and so he began avoiding them. He began to avoid conversations while walking, *“because that's what I was doing when I got shocked.”* He also experienced several spontaneous attacks without a clear trigger.

Due to continued distress from these symptoms, the patient established his first contact with a mental health provider 3 years and 9 months after receiving his first shock. He began counseling treatment with a licensed clinical social worker, every 2 weeks, for a total of 6 visits. He learned basic relaxation techniques including deep abdominal breathing. Other elements of the treatment, in the patient's words included: “*He told me how to deal with the shame of the event, by telling people around me what could happen, what they should do. He told me that if I told more people about it, I wouldn't be as concerned about the public spectacle.”* The patient did not receive any psychiatric evaluation or other forms of treatment.

A week and half prior to psychiatric evaluation at UCLA, and nearly 4 years after his initial SCA, the patient received his fifth set of ICD shocks while at work. During a meeting he had a *“premonition”* and began to feel anxious about being shocked. He began to feel *“not good,”* felt his heart beating faster and *“I knew that a shock was coming.”* After receiving his third shock, he told a coworker to call emergency medical services. He received a series of 13 shocks over a period of minutes. He recalls that when the paramedics arrived and administered IV lorazepam, the shocks seemed to stop. He was again hospitalized for medical evaluation. According to his wife, he would often receive intravenous lorazepam for anxiety, particularly prior to his physicians' arrival for morning rounds, and was discharged with a prescription to take this on an as needed basis.

Based on the pattern of recurrent ICD shocks, the patient was referred by his cardiologist for consideration for BCSD at UCLA. He underwent this procedure the day after his psychiatric evaluation.

### Past medical and surgical history

In addition to experiencing SCA, the patient had been diagnosed with a polymorphic ventricular tachycardia, undergone a dual lead ICD implantation. Surgical procedures included cardiac catheter ablation for PVCs, atrial lead and generator replacement, right shoulder dislocation with subsequent shoulder surgery, and appendectomy.

### Social, family, educational, occupational, and substance use history

The patient received a bachelor's degree in computer engineering and was currently employed as an engineer in a hardware design firm. There was no history of developmental disabilities. He was married, with two children. He denied any history of abuse or arrests. In reference to his early social life, he described himself as an *“awkward teenager,”* whose friendships were mainly with *“outcast kids.”* The patient reported a brief period of marijuana use in college, but otherwise denied any regular alcohol, tobacco or illicit substance use. Family history was notable for a myocardial infarction (paternal grandfather), and alcohol dependence (uncle). There was no other family history of psychiatric illness, and no family history of suicide.

### Medications

At the initial evaluation, the patient was prescribed the following medications: lorazepam 1mg BID prn (patient reported using 2 mg BID for the prior 10 days), metoprolol 10 mg BID, verapamil 120 mg BID, and aspirin 81 mg daily.

### Diagnostic impression and clinical severity

After undergoing a structured clinical interview utilizing MINI International Neuropsychiatric Interview (Sheehan et al., 1998), the patient met DSM IV-TR diagnostic criteria for Post-Traumatic Stress Disorder, Panic Disorder with Agoraphobia, and Social Anxiety Disorder. There was no evidence of axis II pathology. Symptom severity was assessed using several quantitative clinical measures. These indicated “severe” levels of general anxiety (via Beck Anxiety Inventory, BAI (Beck and Steer, 1990), “markedly ill” levels of panic (via Panic Disorder Severity Scale, PDSS) (Furukawa et al., 2009), and borderline clinical depression (via Beck Depression Inventory, BDI) (Beck and Steer, 1993) (Table 1).

**Table 1:** **Psychiatric symptom ratings for Case 1 during 1 year follow-up**.

**Scale**	**Pre-BCSD**	**1 month post**	**3 months post**	**6 months post**	**9 months post**	**1 year post**
BDI (max 63)	18^*^		14^*^	17^*^	10	10
BAI (max 63)	27^*^	27^*^	13^*^	6	2	4
PDSS (max 28)	21^*^	16^*^	13^*^	8^*^	6	2
PCL civilian (range: 17–85)			33	35	31	46^*^
SCS (max 52)				30^*^	16	15
FSAS (range: 10–50)						32^*^

* Indicates scores in a clinically significant range based on scoring criteria for each instrument. BDI, Beck Depression Inventory; BAI, Beck Anxiety Inventory; PDSS, Panic Disorder Severity Scale; PCL, PTSD Checklist—Civilian version. SCS, Shock Catastrophizing Scale; FSAS, Florida Shock Assessment Scale.

### Treatment course

In an attempt to reduce susceptibility to recurrent ventricular tachycardia, partial sympathectomy via BCSD has been applied in selected treatment refractory patients at UCLA (Ajijola and Shivkumar, 2012; Ajijola et al., 2012; Vaseghi et al., 2012, 2013). Bilateral cardiac sympathetic lesioning decreases sympathetic efferent noradrenergic innervation to the heart, and putatively mitigates the pro-arrhythmic effects of sympathetic innervation (Ajijola et al., 2012). The patient underwent this procedure, consisting of a resection covering the lower half of the stellate ganglion and the bodies of sympathetic chain from T2 through T4. He has been followed for approximately 1 year.

Immediately after recovering from the surgery, the patient reported continued episodes of anxiety, including one nocturnal panic attack. He continued utilizing lorazepam 2 mg daily as needed for anxiety, but during these episodes noted “*I couldn't feel my heart racing*.” After 2 weeks, he continued experiencing anxiety that “*comes and goes*.” He was still using lorazepam as needed, approximately every 2–3 days. He had not experienced any further panic attacks.

**Figure 1:**
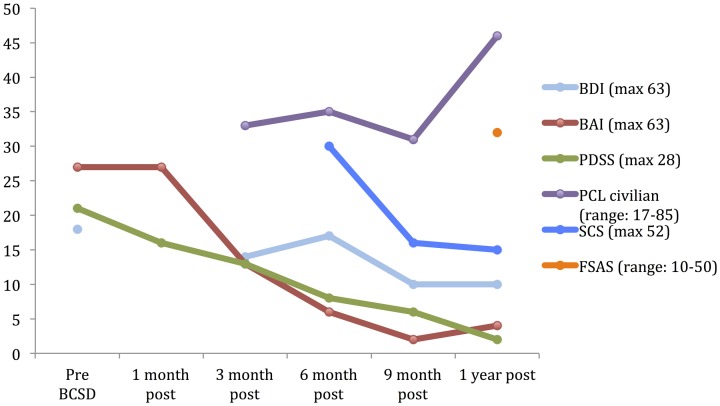
**Time course of psychiatric symptom rating for case 1 during 1 year follow up.** BDI, Beck depression Inventory; BAI, Beck Anxiety Inventory; PDSS, panic Disorder Severity Scale; PCL, PTSD Check list; SCS, Shock Catastrophizing Scale; FSAS, Florida Shock Anxiety Scale. Note the increase in reported PTSD symptoms (via PCL civilian) at 1-year anniversary of worst shock episode, accompained by a moderate score on the Florida Shock Anxiety Scale (typical mean for ICD patient = 15.2, standard deviation = 6.5; from Ford et al., 2012).

After 1 month, the patient had returned to work and reported “*I'm starting to feel better*.” However, he avoided his first work meeting by calling in from home, and still endorsed “*a lot of anxiety*” related to being in situations where he had been shocked previously, including work. When he did eventually attend a work meeting with a colleague, he denied experiencing anticipatory sensations as before, including the absence of a rapid pulse, causing the patient to spontaneously note *“I have noticed differences in how my body reacts.”* When referring back to his home vital sign monitoring records, he reported that his average heart rate had decreased from the 80's to 65. Quantitative examination of his psychiatric symptoms at this time compared to prior to BCSD revealed an unchanged level of general anxiety (via BAI) and slightly decreased intensity of panic (via PDSS) (Figure 1). Based on these continued symptoms, he was referred for cognitive behavioral therapy (CBT) with a PhD psychologist specializing in treatment of PTSD.

At 1 month he reported experiencing a single atypical shock. In the patient's words: *“when leaving work today I was fixing something on the floor. When I stood back up I did not feel right. Not dizzy just felt that something was not right. I took a sip of water to try and calm myself but the feeling did not go away. I walked downstairs and the sensation got stronger. Then I got a single shock. Seemed more mild than others. After the shock I had a real difficult time calming my body down as with prior incidents. Despite this I did not receive a cascade of shocks. Perhaps the benefit of the procedure—stopping my body reaction from impacting the ability of the heart to return to normal rhythm. Despite my panic, I only received a single shock. In the past my inability to calm myself down would result in multiple shocks usually until EMS arrived and administered a calming medication.”* Analysis of his ICD data showed a fast ventricular tachycardia that was different from previously observed ventricular fibrillation. It was felt that this could be due to post-lesion cardiac remodeling. He was started on amiodarone 200 mg BID.

Three months after undergoing BCSD, the patient reported he was *“doing better.”* He was receiving weekly psychotherapy via CBT and had completed 8 sessions. He was learning *“more awareness of what anxiety is and what a panic attack is.”* He was also learning different strategies to cope with and manage his anxiety, including advanced deep breathing techniques and guided pre-recorded audio meditations. He had not started exposure and response prevention, a common and effective behavioral component of CBT, but had begun discussing this approach with his therapist. He reported experiencing continued panic attacks 1–2 per week, but felt these were less severe, and of shorter duration (1–2 min in duration vs. 10 min). In addition, he continued to note *“I don't feel my heart rate like I did during anticipation of shock. I also don't feel PVCs as much as I used to.”* Although he reported walking more, he was still avoiding most meetings by calling in sick, and avoiding going to the gym. Due to his continued symptoms he had been referred to a psychiatrist, had been prescribed a low dose of an SSRI, sertraline, initially 12.5 mg daily and up to 25 mg daily at the time of contact. He continued taking lorazepam on an as-needed basis, but at a lower dose of 1 mg daily three to four times per week. Quantitative review of his symptoms now demonstrated a nearly 50% drop in general anxiety down to a ‘mild’ level (via BAI), a continued decrease in panic severity (via PDSS), and a small but clinically insignificant decrease in depression (via BDI; Figure 1). He had not experienced any further shocks, and no further ICD events were detected.

Six months after undergoing BCSD, the patient continued receiving weekly psychotherapy. He had begun conducting prolonged exposures, a common treatment for psychotherapeutic treatment of PTSD, tailored toward the experience of being shocked. These pertained to *“the memories of the two most significant shock events that have been very difficult for me to think or talk about. I discuss the memory in detail and record it on my phone and then listen to the recording multiple times. It has helped a lot and I no longer have a significant reaction to thinking about those memories.”* He stated he was no longer avoiding going to the gym, and was no longer avoiding individual meetings at work. He continued to express concern about attending group meetings. Sertraline had been increased to 150 mg daily. He noted difficulty *“to differentiate the benefits of the sertraline and [psychotherapy].”* He had not experienced any further shocks, and no further ICD events were detected. In reviewing his progress, he spontaneously noted, *“all in all, I have improved significantly from last fall and I can see a day when I will return to my prior self.”* Quantitative review of his symptoms now demonstrated a nearly 50% drop in general anxiety (via BAI) and a continued decrease in panic severity (via PDSS), with a small but clinically insignificant increase in depression (via BDI; Figure 1).

Nine months after undergoing BCSD, the patient reported *“I have been feeling a lot better lately.”* He continued seeing his therapist but on a slightly decreased basis, weekly to bimonthly. His sertraline had been increased to 200 mg daily. He continued to refrain from utilizing lorazepam. He had not experienced any further shocks, and no further ICD events were detected. Quantitative symptom review revealed that most of the patient's symptoms had continued declining. His general anxiety level was in the “very low” range, and symptoms of panic were now in the “borderline ill” range (Furukawa et al., 2009). His depression was now in the non-clinical range of “normal ups and downs.”

The patient summarized his progress at 9 months as follows: *“At times not even thinking about my condition. Previously, it was a constant thought - constant polling of my body to make sure I felt ok. There have been days where I have felt completely normal. I'm now getting active again. Starting on the treadmill. A remaining fear is associated with activity and getting my heart rate up. Breathing hard is especially a trigger that I have avoided. The difference now is that I no longer view getting on the treadmill as impossible. I feel like I can do it—gradually. Feeling better in social situations also. Had an impromptu lunch with two colleagues last week. Felt good for the entire lunch and participated in conversation and started some conversations. Previously, I had avoided this type of situation. I did have one episode yesterday that caught me off guard. At the end of a meeting we were talking about general things. We were talking about injuries and one person mentioned my SCA [Sudden Cardiac Arrest]. Everyone there knew about my history but one. I have talked about it recently without issue. However, after I started telling the story of my original SCA I had a very strong sensation—rushing feeling like someone is cranking up the volume inside my body. I tensed up expecting a shock. No shock occurred but I got up and returned to my desk and relaxed to decrease my pulse rate.”*

1 year after undergoing BCSD the patient's anxiety, panic and depression symptoms remained markedly attenuated, and in non-clinical ranges (Figure 1). He continued psychotherapy at a further decreased frequency, once per month, and anticipated discontinuing this soon afterwards. He continued on an unchanged dose of sertraline, 200 mg daily. He no was no longer utilizing lorazapam. Other medications included verapamil 120 mg BID, metoprolol 50 mg daily, and aspirin 81 mg daily. His cardiologist had tapered and discontinued amiodarone 1 week before. He had not experienced any further shocks. Review of his ICD data showed no events over the past 6 months, which was the *“first time that has ever happened since my initial arrest.”* This information was particularly *“surprising,”* as this objective record failed to corroborate the subjective diary records of the exact dates and times when he had perceived the body sensations of an abnormal event during that time period.

In subjectively reviewing his symptoms 1 year after BCSD, the patient noted *“I'm starting to have days were I do not constantly think about whether a shock is imminent. I no longer struggle and avoid social situations.”* He noted the lingering presence of cognitive threat appraisals: *“I do still encounter thoughts of playing out scenarios of what would happen if my defibrillator went off—who would see, what would happen. This happens when I'm in social situations especially when walking. I do very occasionally have episodes of what I call an ‘attack.’ Basically, having a physical sensation that mimics the feeling that I get right before a shock. It is like a rushing feeling—tensing up in my body—bracing for the shock. When that happens I usually quickly excuse myself from the situation and calm down and the ‘attack’ passes.”*

He reported further decreased avoidance of exercise: *“I have started walking on the treadmill on a regular basis. Fast walking. Enough to sweat—about 40 min. However, I make a conscious effort to control my breathing and not let my pulse rate get elevated.”* Despite his increased exercise capacity, at 1 year the patient reported he had sustained a 50 pound weight gain. He also endorsed continued preoccupation with receiving a shock during physical exertion: *“The one remaining aspect of my treatment is dealing with my fear of activity. I'm terrified that elevating my heart rate will result in a shock. Frankly, I don't think I will ever move past this fear. The times I have been shocked my pulse has been high. The procedure effectively lowered my pulse rate. So logic leads me to thinking that if the procedure lowers my heart rate then why would I increase it purposefully.”* Exactly 1 year after his worst shock episode, these preoccupations were reflected by moderately elevated scores on the SCS and FSAS, and clinically significant scoring on the PCL (Figure 1).

### Side effects of BCSD

The patient did not experience Horner's Syndrome, an occasional occurrence with stellate ganglion nerve blocks (Schürmann et al., 2001; Lipov, 2010). This was due to the selectivity of the patient's lesion, which spared the caudal head of the stellate ganglion, a region carrying sympathetic nerve fibers to the eye and face. The patient did report the subsequent absence of sweating of the palms and head, and development of hyperhidrosis of the trunk and legs, which have been described as compensatory responses to the severing of sympathetic nerve fibers to the trunk (Hashmonai et al., 2000; Drott et al., 2002).

### Detailed analysis of anxiety symptoms:

**Figure 2:**
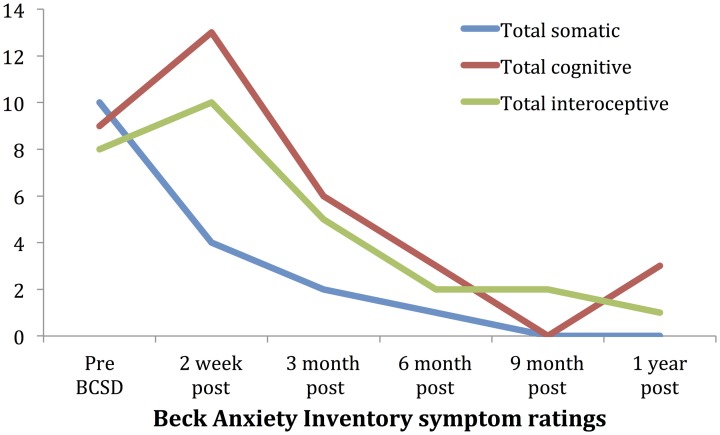
**Time course of Beck Anxiety Inventory somatic, cognitive and interceptive symptoms in Case 1**.

**Figure 3:**
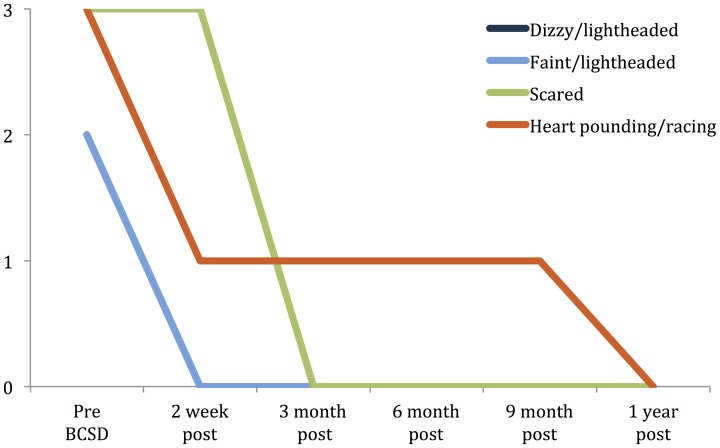
**Time Course of greatest reductions in Beck anxiety symptoms following BCSD in Case 1.** Palpitations and dizziness were the largest reductions reported after BCSD. The largest magnitude reduction overall was feeling scared, which had fully decreased by 3 months, after the patient had begun Cognitive Behavioral Therapy. Scale: 0 = “Not at all.” 1 = “Mildly, but it didn't bother me that much.” 2 = “Moderately—it wasn't pleasant at times.” 3 = “Severely—it bothered me a lot.” Note: ratings for dizzy/lightheaded and faint/lightheaded were identical and overlapping.

Subscale examination of the patient's anxiety revealed several notable patterns (Figure 2, Table 2). During the 2 weeks after undergoing BCSD, the patient reported an overall increase in feeling bothered by several cognitive and interoceptive components of anxiety. The former may have been related to uncertainty about receiving future shocks, and concern about the successfulness of the surgery. The latter may have been related to post-lesion remodeling within the autonomic nervous system that can result in the symptoms of heat sensations, flushing and difficulty breathing reported by the patient. Despite these increases, the patient reported decreases in feeling bothered by dizziness and lightheadedness, and a reduction in feeling bothered by the experience of his heart pounding and racing (Figure 3). Both of these decreases appeared clinically significant, i.e., the symptoms decreased from the maximum score (“severely—it bothered me a lot”) to a mild score (“mildly but it didn't bother me much”). Furthermore, these reductions were sustained throughout the year. In the case of heartbeat sensations, at 1 year the patient reported a complete cessation of symptoms (Figure 3). The only other symptom that declined with a similar magnitude was the feeling of being scared, which decreased entirely between 2 weeks and 3 months after the procedure. Of note, this timeframe corresponded to the initiation of CBT, and formation of a therapeutic bond with his psychologist. It seems unlikely this drop was due to pharmacotherapy, as the patient was no longer utilizing benzodiazepines at that time and had only recently initiated a subtherapeutic dose of sertraline (25 mg daily).

**Table 2:** **Somatic, cognitive and interoceptive anxiety symptoms in Case 1 during 1 year follow-up, from the Beck Anxiety Inventory (BAI)**.

**BAI subscale**	**Pre BCSD**	**2 weeks post**	**3 months post**	**6 months post**	**9 months post**	**1 year post**
**SOMATIC**						
Numbness/tingling	2	2	0	0	0	0
Leg wobbliness	1	0	0	0	0	0
Dizzy/lightheaded	2	0	0	0	0	0
Unsteady	1	0	0	0	0	0
Hands trembling	1	2	2	1	0	0
Shaky/unsteady	1	0	0	0	0	0
Faint/lightheaded	2	0	0	0	0	0
Total somatic	10	4	2	1	0	0
**COGNITIVE**						
Can't relax	0	2	1	1	0	1
Fear worst	0	3	2	1	0	0
Terrified/afraid	2	2	1	0	0	0
Nervous	2	2	0	0	0	1
Fear dying	0	0	0	0	0	0
Scared	3	3	0	0	0	0
Losing control	2	1	2	1	0	1
Total cognitive	9	13	6	3	0	3
**INTEROCEPTIVE**						
Hot	2	3	1	0	0	1
Heart pounding/racing	3	1	1	1	1	0
Choking	0	0	0	0	0	0
Difficulty breathing	1	2	1	0	0	0
Indigestion	0	1	1	0	1	0
Flushed face	2	2	1	0	0	0
Hot/cold sweats	0	1	0	1	0	0
Total interoceptive	8	10	5	2	2	1

Scale: 0, “not at all.” 1, “mildly, but it didn't bother me that much.” 2, “moderately—it wasn't pleasant at times.” 3, “severely—it bothered me a lot.”

The patient also demonstrated a decline in shock related catastrophic thinking (via SCS) between 6 and 9 months post-lesion. This timeframe corresponded to completion of the majority of his CBT visits, and also to the titration of sertraline to a therapeutic dose. Given the concomitant nature of both interventions, is therefore difficult to distinguish which was more likely responsible for the decrease in catastrophizing symptoms during that timeframe.

With regard to symptoms of PTSD assessed via PCL, the patient demonstrated low-moderate scores between 3 and 9 months, which were in the non-symptomatic range via scoring criterion 2 (Blanchard et al., 1996). Exactly 1 year after the patient's last episode of ICD shock storm, the patient's PCL score increased into the symptomatic range. Similarly, at 1 year the patient's score on the FSAS was in the moderate range. Subsequent inquiry revealed that the patient was quite aware of this epoch, and had been thinking about his last shock episode during the days and weeks leading up to this anniversary event. He also explained that he had recently began pushing the limits of his exercise comfort level as a part of his exposure and response prevention psychotherapy, which is known to temporarily increase anxiety levels (Craske and Barlow, 2007). It is possible that his FSAS score could have been decreased (or elevated) prior to this time period, but this remains speculative as this data was unavailable for earlier time points.

## Case 2

### History of present illness

The patient was a 23 year old female with a history of recurrent syncope and suspected seizure disorder who experienced a SCA associated with loss of consciousness while at work during a meeting. She was externally defibrillated by members of the local fire department, and taken to a local hospital where she was found to have a prolonged corrected QT interval of 560 ms. She was diagnosed with idiopathic ventricular fibrillation vs. possible long QT syndrome. Five days after presentation, a dual chamber single coil ICD (Medtronic Inc., St. Paul, MN) was placed for secondary prevention, and she was discharged following a 1 week hospitalization. Three months later, she experienced her first ICD shock. She was sitting down, and began to feel lightheaded, then lost consciousness. She was shocked a total of three times, though she does not remember this. Upon waking, she noticed a pain in the anterior chest radiating to her arm. Interrogation of her ICD revealed she had also received an appropriate shock for an episode of ventricular fibrillation while asleep several days earlier, though she had no memory of this. Device interrogation revealed that the patient had exhibited antitachycardia pacing during defibrillator charging, which had disorganized the ventricular tachycardia into polymorphic ventricular tachycardia and ventricular fibrillation. She was hospitalized for defibrillator threshold testing, in order to ascertain the likelihood that her ICD could successfully terminate a sustained ventricular tachyarrhythmia. Unfortunately, due to a high defibrillation threshold, 6 attempts to elicit ventricular tachycardia termination were unsuccessful. To decrease the risk of recurrent ventricular arrhythmias requiring ICD shocks, the patient was referred for consideration of BCSD.

### Past medical history

Notable for recurrent syncopal episodes with loss of consciousness dating back to high school. On one occasion she may have been noted to bite her tongue, prompting a diagnosis of a seizure disorder, and prescription of phenytoin. There was no history of neurological workup including electroencephalogram (EEG). Nine months prior to first SCA, the patient had a successful vaginal delivery without complications.

### Social, family, educational, occupational, and substance use history

The patient was employed as an assistant restaurant manager. She was married with a 1 year-old daughter. She had received an associates degree in culinary arts. There was no family history of psychiatric illness or cardiovascular disease or syncope or sudden death. No history of substance abuse.

### Medications

At the initial evaluation, the patient was prescribed the following medications: Metoprolol 25 mg BID, Phenytoin 300 mg qHS and KCL 10mEq daily.

### Diagnostic impression and clinical severity

After undergoing a structured clinical interview utilizing MINI International Neuropsychiatric Interview, the patient met DSM IV-TR diagnostic criteria for an adjustment disorder with mixed anxiety and depressed mood. We assessed the degree of severity of the patient's symptoms using several quantitative clinical measures. These indicated “severe” levels of general anxiety (via BAI) and borderline clinical depression (via Beck Depression Inventory, BDI) (Beck and Steer, 1993) (Table 3).

**Table 3:** **Psychiatric symptom ratings for Case 2 during 1 month follow-up**.

**Scale**	**Pre BCSD**	**1 month post**
BDI (max 63)	19^*^	5
BAI (max 63)	27^*^	15^*^
PCL civilian (max 85)	38^*^	35^*^
SCS (max 52)	28^*^	7

* Indicates scores in a clinically significant range based on scoring criteria for each instrument. BDI, Beck Depression Inventory; BAI, Beck Anxiety Inventory; PDSS, Panic Disorder Severity Scale; PCL, PTSD Checklist—Civilian version. SCS, Shock Catastrophizing Scale; FSAS, Florida Shock Assessment Scale.

### Treatment course

The patient underwent a neurology consultation including EEG, to evaluate the possibility of a seizure disorder. The EEG was conducted with the patient in the awake and asleep states. Hyperventilation and photic stimulation were not performed as activating maneuvers, and no sedation was given. While maximally awake, the posterior dominant rhythm was 9–10 Hz, which was symmetric, synchronous and reactive to eye opening and eye closure. Three brief periods of high amplitude theta activity (4–7 Hz) were captured and reported as paroxysmal in nature. They appeared generalized, without a focal cortical distribution, lasting approximately 1–2 s. These slowings occurred in the wakeful and/or drowsy state, with no evidence of epileptiform behaviors at the time. They were interpreted as potentially consistent with physiological drowsiness. In one instance the slowing appeared notched, which could indicate possible epileptiform activity. However, as a fast alpha variant was present at the same time, it was considered suggestive against an epileptic etiology. Vertex waves signifying drowsiness were noted later in the recording, but not at the time of the paroxysmal slowing. This was also considered suggestive against an ictal semiology. Overall, the EEG was judged to be normal in wakefulness and stage II sleep, without any clear electrographic features of seizure. No epileptiform behaviors were identified during clinical observation.

Despite the lack of clear electrographic evidence of a seizure disorder the periods of high amplitude generalized slowing and the reported phenomenology of her episodes were felt to be possibly consistent with a seizure disorder. Seizure prophylaxis was continued, but switched from Phenytoin to Levetiracetam 1000 mg BID.

**Figure 4:**
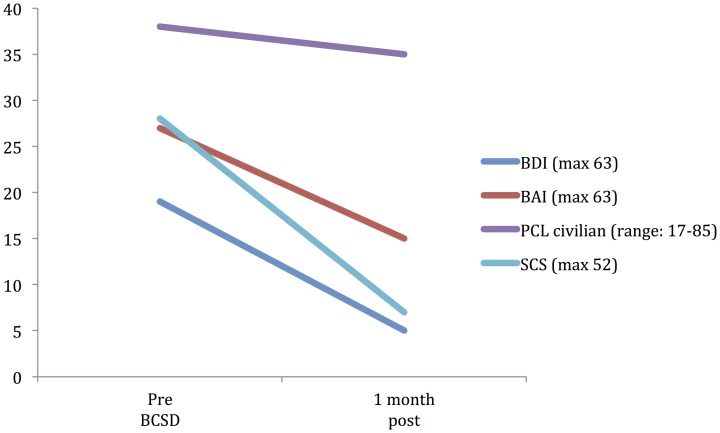
**Time Course of Psychiatric symptom ratings for Case 2 during 1 month follow up**.

After further consultation, the patient elected to undergo BCSD. The resection covered the lower half of the stellate ganglion and the bodies of sympathetic chain from T2 through T4. Discharge medications included Levetiracetam 1000 mg BID and Propranolol 40 mg TID. Evaluation of the patient's symptom scores at 1 month post-BCSD revealed clinically significant decreases in depression (via BDI), anxiety (via BAI) and shock catastrophizing (via SCS) (Figure 4). Clinical evaluation at 1 month post-BCSD, revealed that she had not experienced any further shocks, and no further ICD events had been detected. The patient the patient reported her mood and anxiety symptoms were much improved. She declined the recommendation to seek further psychiatric follow up, and as she was subsequently lost to follow-up no further psychometric data are available.

### Autonomic testing

#### Psychophysiological measures

Continuous measures of skin conductance level (SCL), systolic blood pressure (SBP), and diastolic blood presssure (DBP) were recorded during psychophysiological assessment using Biopac MP150 hardware and Acqknowledge 4.3 software (Biopac, Santa Barbara, CA). Two isotonic 1-cm diameter gel electrodes were attached to the thenar and hypothenar eminence of the subject's nondominant hand to assess SCL. This tonic measure of galvanic skin resistance was selected as a measure of sympathetic arousal due the prolonged time intervals examined (Naqvi and Bechara, 2006). Blood pressure was continuously assessed using the CNAP 500 Monitor (CN Systems, Graz, Austria) with finger cuffs, which were placed between the proximal and distal interphalangeal joints of the third and fourth digits of the subject's nondominant hand. Heart rate was not examined due to the presence of atrial pacing in both patients, which kept the heart rate constant.

#### Laboratory procedures

The psychophysiological assessment began with a 5-min resting baseline during which the subject was seated in a chair and instructed to remain at rest and minimize movement. Psychophysiological measures (SCL, SBP, and DBP) were assessed during the 5-min baseline. As the pre-BCSD testing occurred in an inpatient setting, the first 2 min of the 5-min baseline period were discarded in order to account for possible environment-related influences on autonomic tone. Data for each measure for the final 3 min was averaged to provide a baseline value for that session. Additional interventions to reduce outside distraction included closing the door of the patient's single occupancy room, and silencing the alarms of monitoring equipment. The resting baseline period was followed by 3 tasks in the following order: a valsalva task, a handgrip task, and a mental arithmetic task.

#### Valsalva task

The valsalva task involved performing two sustained valsalva maneuvers while in a seated position. The subject was given the following instructions, *“During this task, I will place my hand on your abdomen. The goal is to push my hand away as far as you can by protruding your abdomen. Hold this position for as long as you can. Remember to keep breathing while bearing down.”* The procedure was demonstrated by the assessor, practiced once by the subject, and then performed twice by the subject. Each trial period lasted approximately 15–25 s. Psychophysiological measures were assessed during the two valsalva trials, and scores on SCL, SBP, and DBP were averaged across the two trials.

#### Handgrip task

The isometric handgrip task involved a hand dynamometer held by the subject's dominant hand. The following instructions were provided before performing the task, *“During this task you will squeeze a hand dynamometer for 2 min at a specific grip strength level with your dominant hand. I will first determine your maximum grip strength level by asking you to squeeze the dynamometer with as much force as possible.”* Following determination of subject's maximum grip strength, the dial on the dynamometer was placed at 30% of the maximum grip strength, and the subject was instructed to squeeze the grip at that level for 2 minutes. Values on SCL, SBP, and DBP were averaged across this 2-min period.

#### Mental arithmetic

The mental arithmetic task was selected as a cognitive stressor and adapted from the Trier Social Stress Task (Kirschbaum et al., 1993). This modified protocol consisted of a 5-min period during which the subject was instructed to consecutively subtract by 13's starting at 1022, in front of two experimenters. The subject was told to perform this task aloud as quickly and accurately as possible, was corrected each time an error was committed, and then told to start over at 1022. Mean SCL, SBP, and DBP were assessed across the 5-min period.

#### Data processing

Acqknowledge software, version 4.3, was used to process all data collected during the psychophysiological assessment sessions. Using Acqknowledge, SCL was averaged across the pre-specified 2 min resting baseline and specified periods during each task described above. The Acqknowledge blood pressure classifier analysis feature was used to identify SBP and DBP at each heartbeat. Means for SBP and DBP were determined for resting baseline and for specified periods during each task. This study was approved by the Unversity of California Los Angeles Institutional Review Board, and all participants provided informed consent prior to participation.

#### Results

**Figure 5:**
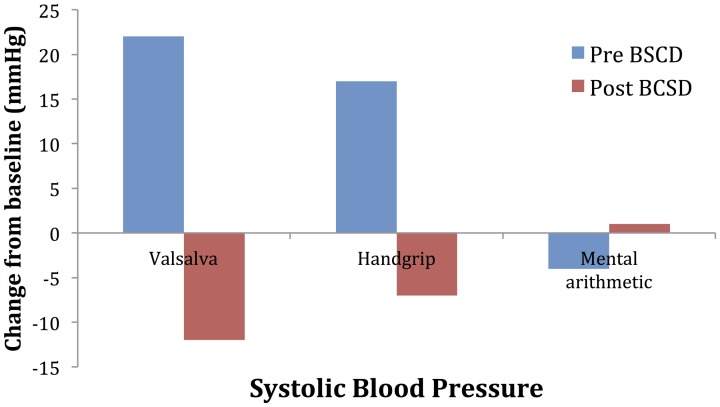
**Mean changes in systolic blood pressure from baseline during autonomic challenge before and after bilateral cardiac sympathetic decentralization (BCSD) in Case 2**.

**Figure 6:**
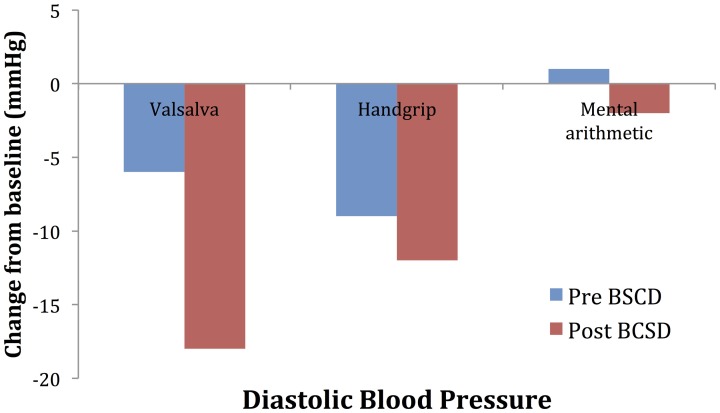
**Mean changes in diastolic blood pressure from baseline during autonomic challenge before and after bilateral cardiac sympathetic decentralization (BCSD) in Case 2**.

**Figure 7:**
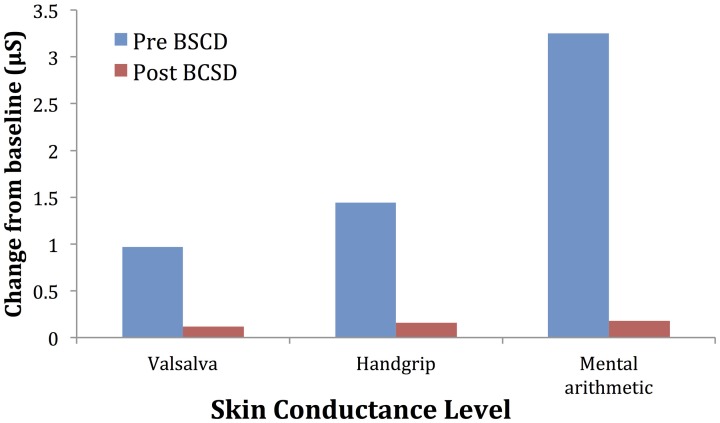
**Mean changes in skin conductance level from baseline during autonomic challenge before and after bilateral cardiac sympathetic decentralization (BSCD) in case 2. μS, microsiemens**.

Mean values of the autonomic testing in Case 2 are displayed in Table 4. Figures 5–7 show changes in each measure from the resting baseline value pre- and post-BCSD. Compared with before BCSD, the patient demonstrated clear declines in baseline adjusted systolic and diastolic blood pressure response for the valsalva and handgrip tasks, but less so with the mental arithmetic stressor, which did not show robust changes at either time point. Comparing pre- vs. post-BCSD, the patient demonstrated prominent declines in baseline adjusted SCL for all tasks (Figure 7).

**Table 4:** **Mean measures of systolic blood pressure (SBP), diastolic blood pressure (DBP) and skin conductance level (SCL) during baseline, valsalva maneuver, isometric handgrip and mental arithmetic stressor, before and after BCSD in Case 2**.

**Pre/ Post**	**Task**	**Normal Beats Count**	**PVCs Count**	**SBP mmHg**	**DBP mmHg**	**SCL μS**
Pre	Baseline	309	0	114	79	1.31
Post	Baseline	305	0	118	85	0.23
Pre	Valsalva	47	0	136	73	2.28
Post	Valsalva	30	0	106	67	0.35
Pre	Handgrip	134	0	131	70	2.75
Post	Handgrip	129	0	111	73	0.39
Pre	Arithmetic	310	0	110	80	4.56
Post	Arithmetic	325	0	119	83	0.41

PVC, premature ventricular contraction; μS, microsiemens.

## Discussion

To our knowledge, Case 1 represents the first reported synergistic combination of BCSD with multimodal psychiatric treatment resulting in successful management of anxiety related to recurrent ICD shocks. The combination of BCSD, cognitive behavioral psychotherapy and pharmacotherapy was sufficient to attenuate the patient's clinical symptoms and maladaptive behaviors, with resulting improvements in work, home and role functioning that were maintained at 1 year. Despite these clinically significant improvements, not all of the patient's symptoms resolved. He continued to experience cognitive threat appraisals on a frequent basis, although these were no longer incapacitating. He also continued to avoid some activities such as vigorous exercise or sexual activity, due to concerns of incurring another shock.

With Case 2, we examined the effect of BCSD on emotional state and autonomic tone. To assess autonomic tone, we chose tasks that have been previously shown to directly activate the medial visceromotor network, which includes the ACC and medial prefrontal cortex (Shoemaker et al., 2012). Compared with before BCSD, we observed robust attenuated sympathetic responses to the valsalva and handgrip tasks, but less so with the mental arithmetic stressor. The patient's lack of autonomic response to this latter task at both time points could explained by several factors, including failure to elicit mental stress due to decreased task difficulty, decreased effort (Brehm and Self, 1989) or due to low trait anxiety in the patient (Gramer and Saria, 2007). Reductions in autonomic responses have been reported following partial sympathectomy in patients with familial hyperhidrosis, including decreased systolic and diastolic blood pressure responses to positional change (Kingma et al., 2002) and during exertion (Nakamura et al., 2002), providing further evidence for the efficacy of this procedure in attenuating sympathetic responses.

The presence of a potential seizure disorder in Case 2 raises interesting questions about the possible impact of an epileptic focus in the brain on the patient's cardiac arrhythmia (Leung et al., 2006; Sevcencu and Struijk, 2010; Marynissen et al., 2012). However, the lack of testing for ictal autonomic semiology during the EEG and lack of examination of the relationship between the paroxysmal slowing and EKG changes limits interpretation of a linking relationship between these conditions.

## Significance

We interpret Case 1's positive response to multidisciplinary treatment in the context of our recently proposed “ABC” model of anxiety (Bystritsky et al., 2013). This model describes a set of processes and underlying brain regions that interact in a cyclical manner to produce anxiety that can generate a variety of anxiety disorders, depending on numerous influences including context, genetics, and environment. In the model, A stands for “Alarm,” defined as emotions, sensations or physiological reactions to perceived threatening situations; B stands for “Belief,” defined as the process of attributing meaning or relevance to the experienced emotions and sensations; C stands for “Coping” response, defined as the specific behaviors or mental activity aimed at reducing anxiety and avoiding the perceived danger. In Case 1, after undergoing BCSD the patient reported attenuated Alarm sensations and physiological responses (e.g., decreased heart rate, decreased heartbeat sensation), despite the presence of the same physical and mental triggers. This alone was insufficient to attenuate his anxiety, likely because he continued to impute these triggers and sensations with dangerous meaning (i.e., Beliefs). Once he began engaging in the psychotherapeutic work that CBT requires of patients (particularly relaxation exercises to further decrease Alarm, cognitive restructuring to reorganize the Belief system, and exposure and response prevention to elicit fear extinction learning), he was able to improve his Coping and change the cycle of his anxiety from a maladaptive to an adaptive state. Though this significantly attenuated his anxiety and panic symptoms, he finally achieved an optimum response when this approach was augmented with serotonergic medication. The greater magnitude of Case 1's anxiety symptoms as compared with Case 2 may have been due to a combination of factors including severity (number of shocks received), duration (greater than 3 years vs. less than 1 year), and differences in genetic and environmental predisposition to experiencing anxiety and distress.

In Case 2 we also observed clinically significant decreases in depression, anxiety and shock catastrophizing following BCSD. This data yields a preliminary hypothesis that the BCSD procedure itself may have a positive impact on mood and anxiety in these patients beyond simply decreasing arrhythmias, ICD shock frequency or reducing autonomic responses to mental and physical stressors. Indeed, partial sympathectomy has been applied as a treatment for several decades in medical disorders with high mortality rates (Schwartz et al., 1991), low mortality rates (Hashmonai et al., 2000), as well as for affective disturbances (Drott et al., 2002) (but see Crozier (2003) for a discussion of the downsides of this latter approach). Such invasive approaches should never be undertaken lightly, and should only be considered after careful examination of the potential risks and extensive discussion with a patient who possesses decision-making capacity. Given the life threatening nature of ventricular arrhythmias, the profound dysfunction that can occur following recurrent ICD shocks, and the repeatedly observed links between emotional stress and ventricular fibrillation (Reich et al., 1981; Lampert et al., 2000; Ziegelstein, 2007), further study appears warranted to explore this hypothesis. Longitudinal comparison with patients who do not undergo BCSD (but who also receive CBT and/or medication management) will be an important comparison, in order to better understand the specificity of the interventions employed in both Cases. Additional studies appear warranted to identify which patient based predictors (e.g., psychological testing, psychiatric diagnosis, autonomic responsiveness) will be most helpful in selecting the optimal combination of treatment approaches.

We noticed that focused reassurance by Case 1's cardiologist during the initial implantation stage appeared to attenuate a specific set of symptoms: fear of death during ICD shock. While provision of reassurance is normally considered maladaptive during psychotherapeutic treatment of primary anxiety disorders, this particular form seemed to enhance the patient's coping response when under the acute duress of receiving a train of shocks. In the context of the ABC model, it appears that this information (i.e., knowledge from cardiologist that “ICD shocks will not kill you”) combined flexibly with his Belief system so that the shocks were not interpreted as dangerously, enabling him to remain relatively calm. Although repeated reassurance can quickly lead to maladaptive safety seeking (as in the aforementioned ICD patient who slept in the hospital for fear of leaving and being shocked), we highlight this instance as it is likely reflective of a broader practice when patients are counseled during the pre-implantation process. If this type of intervention is prominently recalled by patients, then perhaps additional psychoeducation interventions can be employed to help facilitate prompter recovery efforts in patients. Such interventions have been proposed in the past (Sears and Conti, 2003), and systematic pre-implantation interventions have recently been proposed by larger organizations such as the American Heart Association (Dunbar et al., 2012). These proposed interventions are supported by randomized controlled trials that provide important empirical evidence that patients with ICDs can benefit from CBT even in the absence of BCSD (Chevalier et al., 2006; Irvine et al., 2011).

In Case 1, we observed a decrease in reported aversive interoceptive sensations, particularly of palpitations, following BCSD. Although this is a preliminary finding restricted to retrospective report in a single patient, this raises interesting teleological questions about the neurophysiological plausibility of such an effect. That is, how would partial sympathectomy result in decreased cardiac interoceptive awareness, and which peripheral and central neural systems are important in this process? Does decreased efferent noradrenergic signaling to the heart following BCSD result in decreased heart rate, stroke volume and pulse pressure, and thereby affect afferent interoceptive signaling to body sensitive brain regions, such as the insula and somatosensory cortex? (Khalsa et al., 2009a,b; Gazzola et al., 2012). Given the prominent cortical representation of cardiovascular impulses during physical and mental stress (Pollatos and Schandry, 2004; Gray et al., 2007), what are the central neural consequences of BCSD? Are visceromotor signals from the ACC and medial prefrontal cortex, key drivers of autonomic efferent signaling (Shoemaker et al., 2012), altered by BCSD? Beyond a role for the stellate ganglion in afferent interoceptive information transmission, how does this region result in the modulation of emotional experience observed in both cases? Does bilateral interruption of these fibers provide a linking mechanism between the observed improvements in mortality (Ajijola et al., 2012; Vaseghi et al., 2013) and brain networks mediating autonomic functioning, stress, and emotional experience (Lane and Jennings, 1995; Critchley et al., 2005; Lane et al., 2011)? Put more simply, when cortical visceral sensory and motor regions are less bombarded by peripheral information, does this “calm” the brain and decrease ventricular excitability? These and many more questions await investigation.

## Conclusion

Collectively, these preliminary findings suggest that an integrative, multidisciplinary cardiovascular and psychiatric approach to treating ventricular arrhythmias and severe recurrent ICD shocks can result in sustained improvements in physical, psychological and ultimately functional status. The relative roles of psychoeducation, CBT, medication management and BCSD deserve further careful consideration and discussion prior to application in a larger scale.

## Conflict of interest statement

The authors declare that the research was conducted in the absence of any commercial or financial relationships that could be construed as a potential conflict of interest.

## Appendix

Shock Castastrophizing Scale (SCS). Adapted from the Pain Catastrophizing Scale (Sullivan et al., 1995). Scores range from 0 to 52. Crohnbach's alpha coefficient = 0.914, number of items = 13, number of ICD patients completing = 10.

**Table:**

	**Not at all**	**To a slight degree**	**To a moderate degree**	**To a great degree**	**All the time**
I worry all the time about whether the shock will end	0	1	2	3	4
I feel I can't go on	0	1	2	3	4
It's terrible and I think it's never going to get any better	0	1	2	3	4
It's awful and I feel that it overwhelms me	0	1	2	3	4
I feel I can't stand it anymore	0	1	2	3	4
I become afraid that the shocks will get worse	0	1	2	3	4
I keep thinking of other painful events	0	1	2	3	4
I anxiously want the shocks to go away	0	1	2	3	4
I can't seem to keep it out of my mind	0	1	2	3	4
I keep thinking about how much it hurts	0	1	2	3	4
I keep thinking about how badly I want the shocks to stop	0	1	2	3	4
There's nothing I can do to reduce the intensity of the shocks	0	1	2	3	4
I wonder whether something serious may happen	0	1	2	3	4

### Shock catastrophizing scale

We are interested in the types of thoughts and feeling that you have when you think about being shocked by your defibrillator. Using the scale, please indicate the degree to which you have these thoughts and feelings when you anticipate being shocked.
